# Clinical Significance of Molecular Subtypes in Western Advanced Gastric Cancer: A Real-World Multicenter Experience

**DOI:** 10.3390/ijms24010813

**Published:** 2023-01-03

**Authors:** Massimiliano Salati, Michele Ghidini, Matteo Paccagnella, Luca Reggiani Bonetti, Alessandro Bocconi, Andrea Spallanzani, Fabio Gelsomino, Francesca Barbin, Ornella Garrone, Bruno Daniele, Massimo Dominici, Antonio Facciorusso, Angelica Petrillo

**Affiliations:** 1 Division of Oncology, Department of Oncology and Hematology, University Hospital of Modena, 41121 Modena, Italy; 2 PhD Program Clinical and Experimental Medicine, University of Modena and Reggio Emilia, 41121 Modena, Italy; 3 Oncology Unit, Fondazione IRCCS Ca’ Granda Ospedale Maggiore Policlinico, 20122 Milan, Italy; 4 Translational Oncology Fondazione Arco Cuneo, 12100 Cuneo, Italy; 5 Department of Pathology, University of Modena and Reggio Emilia, 41121 Modena, Italy; 6 Medical Oncology Unit, Ospedale del Mare, 80147 Naples, Italy; 7 Gastroenterology Unit, Department of Surgical and Medical Sciences, University of Foggia, 71122 Foggia, Italy

**Keywords:** gastric cancer, molecular subtypes, PD-L1, immunotherapy, EBV, HER2, dMMR

## Abstract

In recent years, the molecular subtyping of gastric cancer has led to the identification of novel clinically relevant biomarkers as well as promising therapeutic targets. In parallel, the advent of checkpoint inhibitors has expanded treatment options beyond conventional chemotherapy. Compelling evidence has shown unprecedented efficacy results for anti-PD1-based therapies in the molecular subgroups of dMMR/MSI-h, EBV+ and PD-L1 CPS+ patients, to the point that these are granted approval for gastric cancer adenocarcinoma (AGC) in several countries. Despite this, cytotoxic chemotherapy remains the only treatment choice for the considerable proportion of biomarkers-negative patients. In this context, little is known about the association between subtypes-defining biomarkers (HER2, MMR/MSI, PD-L1, and EBV) and the efficacy of standard chemotherapy in non-Asian AGC. Here, we aimed to investigate the prevalence, the clinic-pathologic features, and the impact on treatment outcome of clinical molecular subtypes in a new-diagnosed Western cohort of AGC.

## 1. Introduction

Gastric cancer adenocarcinoma (AGC) and gastro-esophageal junction adenocarcinoma (GEA) represent the fifth most common cancer type and the fourth leading cause of cancer-related death worldwide, with over one million new cases and 769,000 estimated deaths in 2020 [1]. The case-to-fatality ratio of GEA is particularly burdensome in Western countries, where late diagnosis and high relapse rates following multimodality treatment make it an incurable disease with a meager prognosis in most cases. Indeed, historical data from both clinical trials and real-world practice reported a median overall survival (mOS) of less than one year for advanced GEA receiving the best chemotherapy combination [2]. The addition of the epidermal growth factor receptor 2 (HER2)-directed agent trastuzumab to a doublet chemotherapy backbone prolonged survival to 14–16 months in HER2-positive cases, though the benefit reduced over time, and the 1-year survival probability is still less than 65% [3]. Since then, incremental yet steady clinical improvements have occurred thanks to a better delivery of cytotoxic agents, the approval of the anti-vascular endothelial growth factor receptor 2 (VEGFR2) agent ramucirumab [4,5], and the oral trifluridine tiparicil [6] in second- and third-line settings, respectively, as well as the early implementation of simultaneous care in the forms of palliative therapy and nutritional support [7,8]. As a result, a growing proportion of patients with advanced disease remains fit to receive multiple lines of treatment (continuum of care) that favorably affect the patient’s outcomes [9].

More interestingly, in the last few years, the profound biological heterogeneity unveiled, among others, by the 2014 The Cancer Genome Atlas (TCGA) molecular classification [10], has been successfully exploited through the rational development of tailored therapeutic interventions. As such, the phase II expansion-platform type II PANGEA trial showed excellent outcomes by using a biomarker-guided personalized treatment strategy in GEA [11]. Accordingly, the subsets of microsatellite instability-high (MSI-h)/deficient mismatch repair (dMMR) patients as well as the expressers of a high level of programmed death ligand one (PD-L1) have been associated with enriched therapeutic benefit from anti–programmed death-1 (PD-1)-based therapies [12].

Recently, practice-changing results from the phase III CheckMate 649 trial showed a statistically significant and clinically meaningful improvement in OS by the addition of the anti-PD1 agent nivolumab to standard-of-care chemotherapy in newly diagnosed GEA, particularly those who are PD-L1 positive, according to the combined positive score (CPS) ≥ 5 [13]. In this context, little is known about the association between subtypes-defining biomarkers (i.e., HER2, dMMR, PD-L1, Epstein Barr virus (EBV)) and the efficacy of standard chemotherapy in non-Asian GEA. Here, we aimed to investigate the prevalence, the clinic-pathologic features, and the impact on treatment outcome of clinical molecular subtypes in a newly-diagnosed Western cohort of GEA. The impact of subtypes on the efficacy of subsequent single-agent anti-PD1 treatment was also evaluated.

## 2. Results

### 2.1. Patient Characteristics

Out of 205 advanced AGC and GEA, a total of 131 patients fulfilling the inclusion criteria were included in the present study.

In the whole population, the median age at diagnosis of advanced disease was 65 years (range 31–84 years old), with a slightly prevalence of the female gender (54%). Major baseline clinicopathologic features were not significantly different across the molecular subtypes (Table 1).

All 131 tissue specimens were collected from the primary tumor before starting first-line treatment: 37 were resected specimens, while 94 were from endoscopic biopsy specimens. All 131 patients were tested for HER2 and MMR status, whereas the results for EBV and PD-L1 expression were available for 50% (65/131) and 49% (64/131) of patients, respectively. Based on the expression of biomarkers, the prevalence of molecular subgroups was as follows: 6.9% for d-MMR, 3% for EBV+, 20.6% for HER2+ and 70.2% for all-negative cases. PD-L1 expression assessed as CPS was ≥5 in 31% (21/64) of cases. Only one overlapping case was found that was both HER2-positive and EBV-positive.

### 2.2. Treatment Outcomes

Regarding first-line treatment, among 104 HER2-negative patients, 83% were treated with a platinum/fluoropyrimidine doublet, while 10% received a taxane-based triplet, and 7% a monochemotherapy with a fluoropyrimidine. Trastuzumab-based chemotherapy was the preferred option for 81% of HER-2-positive cases.

The response to front-line treatment was not significantly different across the molecular subtypes.

**Figure 1:**
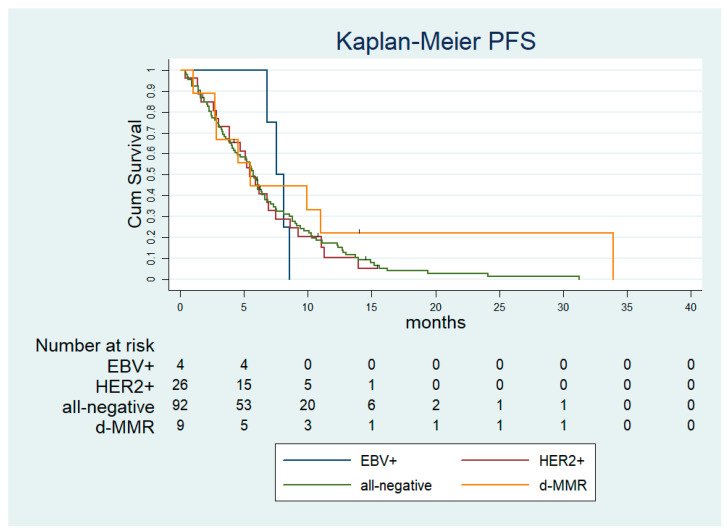
Progression-free survival according to molecular subtypes. dMMR—deficient mismatch repair; EBV—Epstein Barr virus; HER2—epidermal growth factor receptor 2; PFS—progression-free survival.

**Figure 2:**
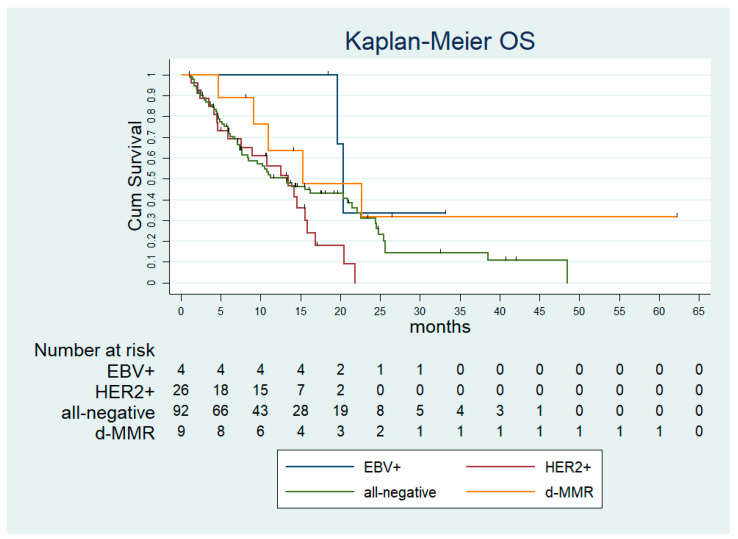
Overall survival according to molecular subtypes. dMMR—deficient mismatch repair; EBV—Epstein Barr virus; HER2—epidermal growth factor receptor 2; OS—overall survival.

**Figure 3:**
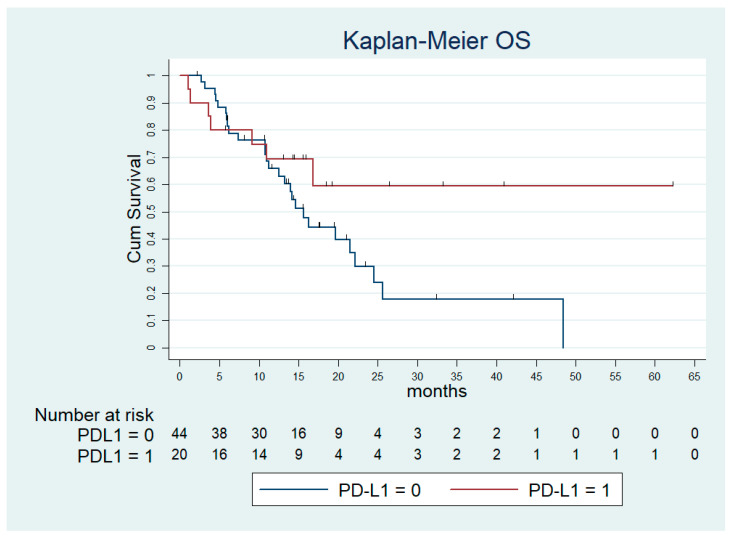
Overall survival by CPS PD-L1 status ≥ 5. Abbreviations: OS—overall survival; PD-L1—programmed death ligand one.

After a median follow-up time of 20.9 months (95% confidence interval [CI]: 14.631–27.205), the median progression-free survival (PFS) and the median OS in the overall population were 5.7 months (standard error [SE]: 0.282; 95% CI: 5.21–6.32) and 13.9 months (SE: 1.849; 95%CI: 10.31–17.55). According to the molecular subtyping, the median PFS was 5.50 months (SE 1.417; 95% CI: 2.73–8.28), 7.54 months (SE 0.65; 95% CI: 6.25–8.82), 5.90 months (SE 0.69; 95% CI: 4.54–7.26) and 5.73 months (SE 0.26; 95% CI: 5.21–6.26), in the d-MMR, EBV+, HER2+ and all-negative subgroup, respectively (Figure 1). The median OS was 15.27 months (SE 6.91; 95% CI: 1.72–28.83), 20.39 months (SE 0.61; 95% CI: 19.18–21.60), 13.47 months (SE 2.52; 95% CI: 8.53–18.41), 13.27 months (SE 2.67; 95% CI: 8.04–18.51) in the d-MMR, EBV+, HER2+ and all-negative subgroup, respectively (Figure 2). Of note, among the subset of PD-L1 CPS ≥ 5 the median PFS was 5.57 months (SE 0.95; 95% CI: 3.70–7.44) and the median OS was not reached (Figure 3).

The proportion of patients receiving second- and third-line treatments were 54% (71/131) and 26% (34/131), respectively. A checkpoint blockade using single-agent anti-PD1 therapy was administered to five patients, of whom one received it as a second-line treatment, while the remaining as a third-line. The median OS for the three d-MMR patients treated with the immune checkpoint inhibitor was not reached and two of them showed a partial response that is still ongoing.

**Figure 4:**
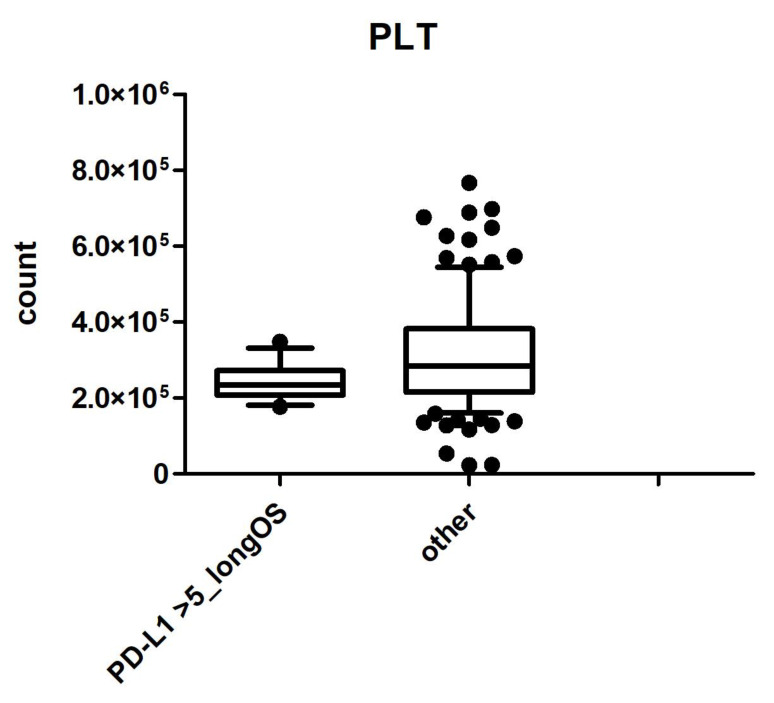
Overall survival by CPS PD-L1 status ≥ 5. Abbreviations: OS—overall survival; PD-L1—programmed death ligand one; PLT—platelets.

With regards to prognostic factors, age, ECOG PS, primary tumor site, disease status, prior surgery, number of metastatic sites, liver, peritoneal and lymph node involvement, treatment intensity, and molecular subtypes were evaluated at the univariate analysis and, among them, prior surgery (*p*-value = 0.04) remained an independent predictor for PFS at the multivariate analysis (Table 2); ECOG PS (*p*-value = 0.02) and prior surgery (*p*-value = 0.02) remained an independent predictor of survival at the multivariate analysis for OS (Table 3). By performing an exploratory analysis, a statistically significant correlation was found between long-term survivor patients with PD-L1 CPS ≥ 5 and low platelet levels (*p*-value < 0.001) (Figure 4).

## 3. Discussion

AGC and GEA have been extensively studied from a molecular point of view in the last years; however, the role of molecular classifications in clinical practice is still under debate. 

In our retrospective series, we aimed to evaluate whether the outcomes of AGC and GEA patients would be affected by the molecular profile in a Western population. We considered four different AGC and GEA subgroups, according to the evaluation of HER-2, microsatellite status and EBV: HER-2+, EBV+, dMMR and all negative. While HER-2+ AGC and GEA accounted for 20% of total cases, in line with Western historical data [14], we recorded a lower prevalence of dMMR (6.9%) and EBV+ (3%) with respect to a previous series that showed an average prevalence of 10% for both subgroups [15,16]. Of note, we did not record overlapping among the subgroups except in one case, which was HER2 and EBV+. Overall, patients with EBV+ disease were the least represented; however, in our series, they had longer median PFS and OS than other subgroups, in line with the fact that EBV + AGC and GEA have unique genomic aberrations, distinct pathological features and a good prognosis with long-lasting and even complete responses to first-line chemotherapy [17,18]. Likewise, patients with d-MMR and with PD-L1 high expression (CPS ≥ 5) had the longest OS. In the latter subgroup, the median was not reached with two patients still under treatment with immune checkpoint inhibitors. Both EBV+ and PD-L1 high-expressed/dMMR patients showed responsiveness to immunotherapy treatment [15] and, undoubtedly, this is a main reason for the better prognosis of these subtypes.

Our results are in disagreement with those previously reported by Kubota et al. [19]. They performed a similar analysis by distinguishing the same four subgroup of patients, but they showed the best PFS in the all negative subtypes (PFS: 4.2, 6.0, 7.5, and 7.6 months in MMR-D, EBV+, HER2+, and all-negative, respectively) treated with a first-line standard chemotherapy; however, the EBV+ subgroup had the best ORR (31%, 62%, 60%, and 49%, respectively). Nevertheless, even if the analysis included a larger cohort (410 patients), we should consider that the study population was entirely from Asia, leading to a lack of power in the comparisons due to the well-known differences among the Asian and Western population for AGC and GEA. Then, Kubota et al., confirmed the benefit of using immunotherapy in patients with dMMR, and showed better outcomes and responses in that subgroup.

Regarding Western populations, to the best of our knowledge, only a single center analysis evaluated the molecular subtype in clinical practice for AGC and GEA [20]. However, unlike our analysis, the study by Martinez-Ciarpagnini assessed only MMR and EBV status, showing 18% dMMR and 6% EBV+ and good tumour-specific survival (TSS) in stages I–III MSI-h AGC and GEA. Then, we should consider that, in our series, checkpoint inhibitors were administered only in five cases and mainly as a third-line, allowing a significant prolongation of OS even in patients pretreated with two lines of therapy. In those cases, a local committee allowed the use of checkpoint inhibitors, namely after gaining approval for each patient.

Regarding later lines of treatment, we showed that 54% and 26% of patients received second- and third-line therapies, respectively. This data is in line with the existing literature [21], underlying the importance of the continuum of care in AGC and GAE concerning an improvement in OS. Interestingly, as concurrent finding, we observed a statistically significant longer survival in patients with PD-L1 CPS ≥ 5 and a lower mean platelet count with respect to the other patients analyzed in our series. On the whole, there is increasing evidence of the suppressive role of platelets on T-cell mediated responses against tumors [21] and some series showed a correlation between the development of thrombocytopenia and improved survival in patients’ treatment with immunotherapy [22]. However, considering the number of patients included in this analysis (only 12 patients with platelet values available), and the lack of validated cut-offs, as well as the controversial results existing in this field [23,24], this finding needs additional further evaluation before being conclusive regarding the role of platelet count or other blood markers [24,25] in GEA.

Nevertheless, our analysis has several limitations. First, it is a retrospective multicenter series with a small sample size. Secondly, only 50% of patients were tested for PD-L1 and EBV status and, therefore, correspondence between dMMR and PD-L1 status is not evaluable. This could be related to the fact that, since immune checkpoint inhibitors in AGC and GEA were not approved and reimbursed as a standard treatment in the clinical practice at the time of the analysis, and at the time of writing as well, testing dMMR and PD-L1 was not mandatory. Additionally, considering that there is a lack of consensus about the methodology used to assess PD-L1 status in GEA, and the type of test is mainly related to the one used in each landmark clinical trial, the test used for PD-L1 was also heterogeneous (e.g., type of antibody).

## 4. Materials and Methods

### 4.1. Patient Selection

Consecutive patients with advanced AGC and GEA and candidates to receive first-line systemic treatment between 1 January 2015 and 1 January 2022 were enrolled at two Italian centers (University Hospital, Modena and Ospedale del Mare, Naples).

The major eligibility criteria were the following: (i) histologically-proven, unresectable, recurrent, locally advanced or metastatic AGC and GEA; (ii) receipt of at least one cycle of first-line chemotherapy (plus or minus the anti-HER2 agent trastuzumab, according to HER2 status); (iii) availability of at least one tested molecular marker in addition to HER2, including MMR, EBV and PD-L1; (iv) Eastern Cooperative Oncology Group Performance Status (ECOG PS) of ≤2 at baseline; (v) adequate bone marrow, hepatic and renal function to receive an active treatment.

Pre-treatment clinic-pathologic and laboratory data were retrieved from the institutional registries of the participating centers through electronic medical records review. 

The study protocol conformed to the ethical guidelines of the 1975 Declaration of Helsinki. Data were collected under the protocol 1186/2018/OSS/AOUMO that was reviewed and approved by the Area Vasta Emilia Nord Ethics committee.

### 4.2. Molecular Characteristics

#### 4.2.1. IHC and ISH Procedures

The tissue samples were fixed in 10% buffered formalin, within 30 min after their surgical removal and were processed after 24 h using the standardized laboratory procedures. From the sections stained with hematoxylin and eosin (HE), we selected blocks containing tumor and that were suitable for immunohistochemistry (IHC) and in situ hybridization (ISH) analysis.

#### 4.2.2. HER2 IHC Tests

We used the HER2 monoclonal antibody (4B5) (Ventana Medical Systems, Inc., Tucson, AZ, USA). HER2 (+3) was assessed as positive, HER2 (2+) as ambiguous and HER2 (+1 oe 0) as negative. IHC Detection Kit was OptiView DAB IHC Detection Kit with OptiView Amplification Kit. Fluorescence in situ hybridization (FISH) test was performed in HER2 (2+) cases.

#### 4.2.3. MMR IHC Tests

For MMR protein detection, we used the VENTANA MMR RxDx Panel that includes the following antibodies: anti-MLH1 (M1) Mouse Monoclonal Primary Antibody, anti-PMS2 (A16-4) Mouse Monoclonal Primary Antibodym, VENTANA anti-MSH2 (G219-1129) Mouse Monoclonal Primary Antibody, and VENTANA anti-MSH6 (SP93) Rabbit Monoclonal Primary Antibody. Stains were performed on BenchMark XT automatic staining (Ventana Medical Systems, Inc., Tucson, AZ, USA). IHC Detection Kit was used for HER2, MLH1, MSH2, MSH6 detections, while the OptiView DAB IHC Detection Kit with OptiView Amplification Kit was used for PMS2.

#### 4.2.4. PD-L1 Test

IHC PD-L1 diagnostic tests were performed on each sample using 22C3 pharmDx (primary anti-PD-L1 murine monoclonal antibody, prediluted, clone 22C3, Dako, Carpinteria, CA, USA), according to the manufacturer’s instructions. The interpretation of the assays was performed by dedicated pathologists who received adequate training. PD-L1 expression in the tumor cell membrane and in the membrane and/or cytoplasm of tumor-associated mononuclear inflammation cells such as lymphocytes and macrophages were evaluated. CPS score was calculated as follows: total number of tumor cells and immune cells (including lymphocytes and macrophages) stained with PD-L1, divided by the number of all viable tumor cells, and then multiplied by 100.

#### 4.2.5. ISH Analysis

The tissue samples were fixed in 10% buffered formalin, within 30 min after their surgical removal and were processed after 24 h using the standardized laboratory procedures. From the sections stained with hematoxylin and eosin (HE), we selected blocks containing tumor and that were suitable for IHC and ISH analysis.

### 4.3. Statistical Analysis

In this study, we evaluated the objective response rate (ORR), defined as the proportion of patients who achieved a complete response (CR) or a partial response (PR), the progression free survival (PFS) and the OS by predefined molecular subtypes and PD-L1 CPS status. Tumor response was assessed in patients with measurable lesions with the Response Evaluation Criteria in Solid Tumors (RECIST) version 1.1.

The PFS was calculated as the interval between the beginning of the first-line treatment until disease progression or death from any cause or the last follow-up visit in the case of censored patients.

The OS was calculated as the interval between the beginning of the first-line treatment until death from any cause or the last follow-up visit in the case of censored patients.

The Fisher exact test was preferred whenever it was possible, in the other cases, the χ2 test was used to compare the baseline characteristics and the response to molecular subtype. The OS and PFS were compared among the molecular subtypes and other characteristics using the Kaplan–Meier method. Additionally, the impact of covariates on the PFS and OS was evaluated using the Cox proportional hazard method and presented as HR with 95% confidence intervals (CIs). Covariates with a known prognostic significance in GEA were tested at the univariate analysis and multivariate analysis levels.

The Fisher exact test and χ2 test were performed with GraphPad 5.0, while the Kaplan–Meier and Cox analyses were performed with IBM SPSS v.24. *p* < 0.05 was considered statistically significative.

## 5. Conclusions

AGC and GEA are a heterogeneous group of diseases and, even if much has already been already performed in the field of molecular characterization of those tumors, little is known about the prognostic and predictive meaning of the molecular classifications. Although survival outcomes did not significantly differ across the molecular subtypes, our analysis suggest that biomarker-defined subgroups of AGC and GEA may be linked to differential treatment response in a Western population. Then, despite the reduced number of patients receiving immune-checkpoint inhibitors, “immunosensitive” subgroups such as dMMR, EBV+ and PD-L1 CPS ≥ 5 showed the best survival outcomes, suggesting a possible prognostic role of this molecular characterization in the management of AGC and GEA. Therefore, HER2, dMMR, EBV and PD-L1 CPS could be useful in clinical practice and should be performed routinely.

## Figures and Tables

**Table 1:** Baseline patients’ characteristics.

		d-MMR N = 9 (6.9%)	EBV+ N = 4 (3.1%)	HER2+ N = 26 (19.8%)	All-Negative N = 92 (70.2%)
Age	Median (range)	69 yrs (54–83)	69.5 yrs (60–74)	61 yrs (35–83)	65 yrs (31–84)
Gender	Male Female	3 (33.3%) 6 (66.7%)	2 (50.0%) 2 (50.0%)	14 (53.8%) 12 (46.2%)	52 (56.5%) 40 (43.5%)
ECOG PS	Median (range)	1 (0–2)	0 (0–1)	1 (0–2)	1 (0–2)
Prior surgery	Yes	4 (44.4%)	1 (25.0%)	7 (26.9%)	25 (27.2)
Primary site	Stomach	5 (55.6)	2 (50.0%)	17 (65.4%)	70 (76.1%)
	Junction	4 (44.4)	2 (50.0%)	9 (34.7)	22 (23.9%)
Disease status	Locally advanced unresectable	3 (33.3%)	2 (50.0%)	5 (19.2%)	11 (12.0%)
	Metastatic	6 (66.7%)	2 (50.0%)	21 (80.8%)	81 (82.0%)
N° metastasis	≥2	6 (66.7%)	0 (0.0%)	13 (50.0%)	52 (56.5%)
Site of metastasis	Hepatic	1 (11.1%)	1 (25.0%)	9 (34.6%)	21 (22.8%)
	Peritoneal	2 (22.2%)	1 (25.0%)	17 (65.4%)	48 (52.2%)
	Lymph node	5 (55.6%)	2 (50.0%)	12 (46.2%)	52 (56.5%)
PD-L1 (>5%) (N = 20)		3 (33.3%)	2 (50.0%)	3 (11.5%)	12 (13.0%)
Response	PR	4 (44.4%)	2 (50%)	10 (37.0%)	35 (38.0%)
	SD	2 (22.2%)	1 (25%)	4 (14.8%)	23 (25.0%)
	PD	3 (33.3%)	1 (25%)	12 (44.4%)	30 (32.6%)

Abbreviations: dMMR—deficient mismatch repair; EBV—Epstein Barr virus; ECOG PS—Eastern Cooperative Oncology Group Performance Status; HER2—epidermal growth factor receptor 2; PD—Progressive Disease; PD-L1—programmed death ligand one; PR—partial response; SD—stable disease.

**Table 2:** Cox proportional hazard model for PFS.

Variable		Univariate			Multivariate	
	HR	95% CI	*p* Value	HR	95% CI	*p* Value
**Age** >70 yrs vs. ≤70 yrs	0.934	0.634–1.377	0.731	1.167	0.717–1.900	0.533
**Sex** male vs. female	0.742	0.517–1.065	0.106	/		
**ECOG PS** **(0–1 vs. 2)**	0.684	0.476–0.982	0.040	0.670	0.445–1.009	0.055
**Primary tumor Site** (Gastric vs. GEJ)	0.729	0.485–1.096	0.129	0.725	0.465–1.131	0.156
**Disease status** (Metastatic vs. Locally advanced)	0.769	0.470–1.258	0.295	1.121	0.588–2.136	0.729
**Prior Surgery** **(Yes vs. No)**	1.575	1.051–2.360	0.028	1.820	1.040–3.188	0.036
**No. of metastatic sites** (>2 vs. other)	0.914	0.638–1.308	0.623	0.893	0.524–1.520	0.676
**Type of metastasis** (Liver vs. other)	1.031	0.682–1.559	0.885	1.307	0.714–2.391	0.385
**Type of metastasis** (Peritoneum vs. other)	0.919	0.641–1.318	0.647	1.155	0.674–1.981	0.600
**Type of metastasis** (Lymph node vs. other)	1.212	0.840–1.749	0.304	1.190	0.709–1.998	0.510
**Regimen** FLOT vs. other	1.646	0.801–3.385	0.175	1.477	0.645–3.378	0.356
**MMR status** (pMMR vs. dMMR)	0.594	0.274–1.287	0.187	0.581	0.239–1.415	0.232
**EBV status** (+ vs. −)	0.965	0.344–2.709	0.946	1.124	0.355–3.555	0.842
**HER2 status** (+ vs. −)	0.926	0.586–1.462	0.741	0.957	0.571–1.603	0.866
**All-negative** (vs. others)	0.884	0.595–1.315	0.554	/		
**PD-L1 CPS ≥ 5** (n = 64)	1.006	0.575–1.758	0.983	/		
**Treatment intensity** (Triplet vs. other)			0.155			0.390
monotherapy	1.875	0.959–3.668	0.066	1.200	0.445–3.234	0.719
doublet	1.181	0.715–1.949	0.516	0.783	0.394–1.555	0.366

Abbreviations: CI—confidence interval; CPS—combined positive score—dMMR—deficient mismatch repair; EBV—Epstein Barr virus; ECOG PS—Eastern Cooperative Oncology Group Performance Status; FLOT—5-fluorouracil, oxaliplatin, docetaxel; GEJ—gastro-esophageal junction; HER2—epidermal growth factor receptor 2; HR—hazard ratio; MMR—mismatch repair; pMMR—proficient mismatch repair; PD-L1—programmed death ligand one.

**Table 3:** Cox proportional hazard model for OS.

Variable		Univariate			Multivariate	
	HR	95% CI	*p* Value	HR	95% CI	*p* Value
**Age** >70 yrs vs. ≤70 yrs	1.093	0.686–1.743	0.708	1.100	0.623–1.942	0.742
**Sex** male vs. female	0.491	0.318–0.758	0.001			
**ECOG PS** **(0–1 vs. 2)**	0.588	0.382–0.905	0.016	0.568	0.354–0.912	0.019
**Primary tumor site** (Gastric vs. GEJ)	0.662	0.407–1.077	0.097	0.650	0.377–1.122	0.122
**Disease status** (Metastatic vs. Locally advanced)	0.494	0.260–0.938	0.031	1.017	0.440–2.353	0.968
**Prior Surgery** **(Yes vs. No)**	2.434	1.441–4.111	0.001	2.298	1.155–4.572	0.018
**N. of metastatic sites** (>2 vs. other)	0.701	0.455–1.080	0.107	0.828	0.444–1.543	0.553
**Type of metastasis** (Liver vs. other)	0.956	0.588–1.554	0.856	1.212	0.601–2.443	0.591
**Type of metastasis** (Peritoneum vs. other)	0.612	0.398–0.943	0.026	0.917	0.497–1.693	0.783
**Type of metastasis** (Lymph node vs. other)	1.344	0.870–2.076	0.182	1.308	0.706–2.422	0.394
**Regimen** FLOT vs. other	1.225	0.534–2.813	0.632	0.817	0.319–2.090	0.673
**MMR status** (pMMR vs. dMMR)	0.513	0.206–1.279	0.152	0.724	0.273–1.922	0.517
**EBV status** (+ vs. −)	1.874	0.445–7.892	0.392	1.673	0.366–7.646	0.507
**HER2 status** (+ vs. −)	0.579	0.348–0.961	0.035	0.572	0.324–1.007	0.053
**All-negative** (vs. others)	1.016	0.646–1.596	0.946	/		
**PD-L1 CPS ≥ 5** (n = 64)	1.972	0.855–4.549	0.111	/		
**Treatment intensity** (Triplet vs. other)			0.024			0.646
monotherapy	3.256	1.393–7.656	0.006	1.636	0.525–5.095	0.396
doublet	1.795	0.919–3.504	0.087	1.149	0.497–2.659	0.745

Abbreviations: CI—confidence interval; CPS—combined positive score—dMMR—deficient mismatch repair; EBV—Epstein Barr virus; ECOG PS—Eastern Cooperative Oncology Group Performance Status; FLOT—5-fluorouracil, oxaliplatin, docetaxel; GEJ—gastro-esophageal junction; HER2—epidermal growth factor receptor 2; HR—hazard ratio; MMR—mismatch repair; pMMR—proficient mismatch repair; PD-L1—programmed death ligand one.

## Data Availability

Not applicable.
